# American Dental Association

**Published:** 1890-09

**Authors:** 


### Editorial.

### AMERICAN DENTAL ASSOCIATION.

This Association convened at the appointed time at Excelsior Springs, Mo. The attendance was large, but not equal to the expectations of the Western members, who had anticipated a gathering equal in numbers to that held at Minneapolis. That this was not so must be ascribed to the financial stringency prevailing throughout that section of the West. The East was poorly represented. This was to be expected. The distance, coupled with the extreme heat prevailing at the time, doubtless had an influence in keeping away many of those accustomed to taking part in these annual gatherings. Notwithstanding these drawbacks, the meeting was one of the most satisfactory that the Association has had in several years. This was mainly owing to the admirable planning of the Committee of Arrangements, in which they successfully avoided the unpleasant experiences at Saratoga last year. They arranged for two meetings a day, morning and evening, giving the afternoon to the National Association of Faculties and National Board of Examiners. This enabled these bodies to accomplish their work without conflict, and gave the members opportunity to take part in the proceedings of the parent organization.

The work of the Association as a whole cannot but be regarded as of superior character. Several of the sections were well prepared, and in one or two instances the reports were exceptionally good. That on "Dental Literature," Dr. C. N. Peirce, chairman, was especially noticeable for the collection of valuable matter and for its suggestive ideas; indeed, it may be regarded as a model in this respect.

The most important action was that of the appointment of a committee to act with the Southern Dental Association for an International Meeting to be held at Chicago in 1893. This action was not taken with the deliberation so essential in matters of this import. It seemed as though a fear existed that delay might arouse opposition; hence the apparently prearranged plan to force it through at a very early period of the meeting. This, if true, was a needless expenditure of effort, as there was, probably, no decided amount of opposition to a meeting in Chicago that year.

There was a marked improvement over recent years, in that politics formed no large part of the thoughts of the members of the Association. It was settled very early that the presidency should go to Chicago, and very properly to Dr. Harlan. This was carried out by a large majority to the satisfaction of all parties. The selection of a place of meeting for next year (1891) was left to the Committee of Arrangements. This course was taken owing to the difficulty in making terms with the railroads. The decision will probably lie between Niagara Falls and Saratoga, although there was a strong desire on the part of many Western members that some place on the Atlantic coast should be selected.

One of the most encouraging features of this meeting was the feeling manifested, privately and publicly, that the time had arrived when the American Dental Association must make a decided change in its management,—that the old mode of organization had had its day of usefulness. The present plan of permanent membership and delegates from local associations was conceded not to be a satisfactory mode of organization. It has taken the members a long time to reach this conclusion, and it is to be hoped that by another year this general feeling may be crystallized into form looking to a radical change in the constitution. No such composite body—a wheel within a wheel—can hope to preserve the confidence of the profession. What is needed is a thoroughly representative association closely connected with State and local organizations in the United States. The objections heretofore raised to this plan have no force, and should not stand in the way of a future reorganization making this Association fully in harmony and closely affiliated with subordinate societies within its jurisdiction. It is, perhaps, too soon to expect that the Southern will be permanently united with the American Dental Association; but that this will be the final result we feel assured. The multiplication of professional organizations is a positive evil, and the unification of scientific work should be the aim of all who desire to see sectionalism banished from professional effort.

The selection of Excelsior Springs proved a very happy one. Some of the weary and overheated travellers were disposed, as they approached it, to regard the place with misgivings, and expressed a fear that a mistake had been made. Further experience proved this hasty conclusion an error.

The company owning this tract with its numerous springs has expended a large sum in beautifying the grounds, naturally well adapted in grove and lawn for this purpose. The large hotel, placed in the midst of these surroundings and provided with all the conveniences considered necessary for our modern civilization,—elevators, electric lights, etc.,—make it difficult to realize that this is located in the county from which Quantrell sallied forth in his raids, and where Jesse James terrorized for so long a period. Western Missouri is a garden-spot, not as yet appreciated by the people at large.

The untiring attentions of the managers of the Excelsior Springs Company made this meeting of the American Association one to be remembered by all who were so fortunate as to be present. Nothing was omitted by them to make the meeting a success, professionally as well as socially; and should it be necessary for the Association to go as far West again, no mistake would be made in selecting this spot in the border-land of Missouri and Kansas.

